# PID1 alters the antilipolytic action of insulin and increases lipolysis via inhibition of AKT/PKA pathway activation

**DOI:** 10.1371/journal.pone.0214606

**Published:** 2019-04-16

**Authors:** Chunyan Yin, Wei hua Liu, Yuesheng Liu, Li Wang, Yanfeng Xiao

**Affiliations:** The Second Affiliated Hospital of Xi'an Jiaotong University, Xi’an, Shan Xi, People’s Republic of China; Northwest University, UNITED STATES

## Abstract

**Purpose:**

The aim of this study was to investigate the effect of phosphotyrosine interaction domain containing 1 (PID1) on the insulin-induced activation of the AKT (protein kinase B)/protein kinase A (PKA)/hormone-sensitive lipase (HSL) pathway and lipolysis.

**Methods:**

Sprague–Dawley rats were fed either chow or a high-fat diet (HFD). The levels of insulin, glycerol, free fatty acids (FFAs) and PID1 mRNA expression were measured in the 2 groups. Furthermore, we examined the role of PID1 in the regulation of the AKT/PKA/HSL cascade and lipolysis in the 3T3-L1 cell line.

**Results:**

Adipose tissue from HFD rats exhibited elevated PID1 expression, which showed a positive correlation with insulin levels and lipolysis. In 3T3-L1 adipocytes, we found that the antilipolytic effect of insulin is mediated by AKT and that phosphorylated AKT results in the promotion of PDE3B expression, the dephosphorylation of PKA and HSL and the suppression of glycerol release. However, overexpression of PID1 and treatment with 1 μM isoproterenol and 100 nM insulin for 24 h resulted in an increased release of glycerol and a noticeable inhibition of AKT phosphorylation, PDE3B expression and the phosphorylation of PKA/HSL in 3T3-L1 cells. In contrast, knockdown of PID1 and treatment with the above reagents inhibited lipolysis and activated the phosphorylation of AKT, which resulted in the dephosphorylation of PKA and HSL.

**Conclusions:**

Our findings indicate that PID1 in adipose tissue increases lipolysis by altering the antilipolytic action of insulin. This suggests that PID1 may represent a new therapeutic target to ameliorate adipocyte lipolysis and hence improve insulin sensitivity.

## Introduction

Obesity is an increasing global health problem that is usually accompanied by insulin resistance (IR) and type 2 diabetes mellitus (T2DM). Elevated serum levels of free fatty acid (FFA) are frequently observed in patients with T2DM. A wide body of evidence suggests that elevated FFA levels are a consequence of inappropriate lipolysis, which is a major etiological factor for IR and T2DM [1–2]. Thus, understanding the mechanism by which impaired insulin suppresses fat cell lipolysis is critical for identifying the underlying defect in resistant adipose tissues and ultimately for developing effective therapeutics.

In addition to regulating glucose metabolism, insulin plays a key role in promoting lipogenesis and inhibiting lipolysis [3].The antilipolytic effect of insulin is believed to involve a reduction in cyclic adenosine monophosphate (cAMP) levels and thus the activity of protein kinase A (PKA). In this model, insulin signaling activates phosphodiesterase3b (PDE3b) via the protein kinase B-mediated phosphorylation of Ser273 [4, 5]. The activation of PDE3B catalyzes the hydrolysis of cAMP, which reduces the cellular level of cAMP. The lowering of cAMP further inhibits PKA activity and thereby results in a decrease in hormone-sensitive lipase (HSL) and lipolysis [6]; however, recent results suggest that PDE3B activity in adipose tissue is substantially reduced in obese patients [7]. Thus, decreased activity of the AKT/PDE3B pathway may contribute to the diminished antilipolytic effect of insulin in obese patients.

PID1 (also referred to as NYGGF4) is a novel gene that was initially isolated and characterized in obese subjects. It is a 1527-bp cDNA containing 753 nucleotides of an ORF (open reading frame) predicting 250 amino acids with a molecular mass of 28.27 kDa[8]. Amino acid sequence analysis revealed that PID1 has a phosphotyrosine-binding (PTB) domain, which can bind to phosphorylated tyrosine residues, impair insulin signal transduction, and lead to obesity-related IR [9]. The binding of insulin to its cell surface protein receptors causes tyrosine phosphorylation, which results in the phosphorylation of insulin receptor substrates on specific tyrosine residues and the activation and recruitment of PI3 kinase and its downstream target, AKT. Given that PID1 is an important protein in AKT signaling [10] and a key player in mediating the antilipolytic effect of insulin, we hypothesized that PID1 may influence the AKT transduction pathway of insulin.

In this study, we examined the effects of PID1 on lipolysis in high-fat diet (HFD)-induced obese rats and further investigated the potential molecular mechanisms that underlie these effects in vitro using 3T3-L1 cells. We present evidence that PID1 alters the antilipolytic effect of insulin by inhibiting the AKT/PKA pathway, which is activated by insulin and leads to lipolysis in obese individuals.

## Methods

### Animal care and treatment schedule

Ninety-six male Sprague–Dawley (SD) rats (age: 3 weeks) were obtained from the Animal Center of Xi’an Jiao tong University and individually housed in a humidity controlled room with a 12 h light/dark cycle. All rats were fed a commercial diet for 1 week. Subsequently, the animals were randomly allocated to one of two dietary groups at a ratio of 1:2, i.e., normal diet [ND (n = 32), 12% kcal fat] and high-fat diet [HFD (n = 64), 60% kcal fat] groups. Eight rats from the ND group and 16 rats from the HFD group were randomly selected, and their body weights were measured at 8, 16, 20, and 24 weeks (w). The experimental protocols were approved by the Animal Care and Protection Committee of Xi’an Jiao tong University.

### Blood biochemistry

The rats were allowed access to food between 8 am and 10am on the day of execution. After feeding, at 10.00 am, all rats were euthanized, and their peripheral and epididymal fat pads were excised and weighed. Total body fat included peripheral and epididymal fat pads. Blood samples were collected to measure the levels of insulin and glycerol by ELISA (Sigma) at 8, 16, 20 and 24 w. Enzymatic assay kits (Applygen) were used to determine serum glucose levels. Samples of adipose tissue were collected to detect the mRNA expression of PID1 by RT-PCR.

### Insulin binding to isolated fat cells

We studied insulin binding using fat cells isolated from rats in the HFD and ND groups at 24 w. Isolated fat cells from peripheral fat pads were prepared by Rodbell's method and suspended in buffer containing 35 mM Tris, 120 mM NaCl, 1.2 mM MgSO4, 2.5 mM KCl, 10 mM glucose, 1 mM EDTA, and 1% bovine serum albumin at pH 7.6 and incubated with ^125^I-insulin and unlabeled insulin in plastic flasks in a 24°C shaking water bath as previously described. Optimal steady-state binding conditions were achieved at 24°C after 45 min of incubation. The binding reaction was terminated as described by Gammeltoft and Gliemann[11] by removing 200-μL aliquots from the cell suspension and rapidly centrifuging the cells in plastic microtubes in which 100 μL dinonyl phthalate oil had been added. The supernatant was then removed, and the cell-bound radioactivity was determined.

### Cell culture

3T3-L1 cells were obtained from the American Tissue Culture Collection (ATCC) and cultured in flasks (25 cm^2^) containing phenol red-free Dulbecco’s modified Eagle’s medium. Differentiation was induced using protocols described elsewhere [12]. When >90% cells were fully differentiated, 200-μL aliquots were placed into 5-mL polypropylene tubes, and 1 μM isoproterenol, a β-adrenergic receptor agonist, and increasing concentrations of insulin (1–100 nmol/L) were added. Adipocytes were incubated for 1 h at 37°C in a shaking water bath (100 rpm), and the glycerol concentration in the cell medium was measured using free glycerol reagent. To block the PKA pathway, the PKA inhibitor H-89 was added 12 h after exposure to 100 nM insulin and 1 μM isoproterenol for 24h; subsequently, the culture medium and cells were separated and stored.

### Oil red O staining and quantification of lipid accumulation

Adipocytes were fixed for 40 min with 10% formalin, washed with PBS, stained for 2 h by complete immersion in a working solution of Oil red O, and exhaustively rinsed with water. Excess water was evaporated by placing the dishes at 37°C. The dye was extracted with 200 μL of isopropyl alcohol per well, and its absorbance was monitored spectrophotometrically at 510 nm. Lipids were quantified using triolein (C18: 1, [cis]-9, Sigma) calibration curves as previously described [13].

### cAMP determination

Differentiated 3T3-L1 adipocytes cultured in 35-mm plates were deprived of serum for 12 h, and the cells were treated with 1μM isoproterenol and different concentrations of insulin for 24h. Adipocytes were then washed with PBS and lysed in 0.1 mM hydrochloric acid. After centrifuging at 2,000 × g for 15 min at 4°C to remove insoluble materials, supernatants were used to measure cAMP contents with a cAMP enzyme immunoassay kit (Amersham).

### Lipolysis measurement

An aliquot of the media (400 μL) was collected, and glycerol release in cell culture medium was determined using a colorimetric method (Sigma). The amount of glycerol was normalized to protein concentration as an index of lipolysis.

### Immunofluorescence

3T3-L1 cells were cultured and differentiated on cover slips, fixed with 4% paraformaldehyde for 20 min, permeabilized with 0.05% Triton X-100 in PBS (15 min), and blocked with 5% BSA in PBST (1 h at room temperature). Staining with the PID1 antibody (1:1000; Santa Cruz, CA, USA) was followed by incubation with AlexaFluor (488)-conjugated secondary antibodies(Jackson), staining with 0.2 μg/mL Nile Red (Sigma) for 5 min, and incubation with 0.1 μg/mL DAPI for 2 min.

### Immunoblot analysis

Cells were lysed in ice-cold RIPA buffer. Total proteins or phosphorylated proteins were extracted as described previously. Protein levels were quantified using the bicinchoninic acid protein assay kit (Pierce, Rockford, IL, USA) in accordance with the manufacturer's instructions. After measuring protein concentration, the samples were mixed with Laemmli sample buffer and subjected to polyacrylamide gel electrophoresis (PAGE) (10% acrylamide) and Western blot analysis. After the electrotransfer of proteins onto a PVDF membrane (Millipore), membranes were incubated overnight at 4°C with continual motion using specific primary antibodies [AKT(ab108385, Abcam, USA, mouse, 1:500), p-AktSer^473^ (ab176657, Abcam, USA, mouse, 1:500), PDE3B (ab42091, Abcam, USA, mouse, 1:1000), PKA (ab75996, Abcam, USA, mouse, 1:500), p-PKAThr^197^ (ab75991, Abcam, USA, mouse, 1:1000), HSL (#4107, CST, USA, mouse, 1:1000), p-HSLSer^563^(#4139, CST, USA, mouse, 1:1000), ATGL (ab109251, Abcam, USA, mouse, 1:1000), and p-ATGLSer^406^ (ab135093, Abcam, USA, mouse, 1:1000)]. The detection of protein–antibody immune complexes was achieved using horseradish peroxidase-conjugated secondary antibodies diluted 1:10000 in PBS with 0.05% Tween. After the addition of the chemiluminescent substrate, films were exposed for 5 min. The bands were detected using an enhanced chemiluminescence detection system (Amersham). β-Actin (Sigma) served as a control. Scanning densitometry was performed by acquisition into Adobe Photoshop (Apple, Inc., Cupertino, CA), and analysis was performed using Quantity One (Bio-Rad system).

### PID1 overexpression and silenced cell culture and treatment

PID1 is a 1527-bp cDNA encoding 250 amino acids with a molecular mass of 28.27 kDa. The sequences of the two cDNA fragments (PID1siRNA, 5′-AAGGTGAATAGACACATT-3′; andnegativecontrol, 5′-GTTCTCCGAACGTGTCACG-3’) were subcloned into the pGPU6/GFP/Neovector to generate an empty expression vector (pGPU6–NC–shRNA) or a PID1-silenced vector (pGPU6- PID1-shRNA). The coding sequence of mouse PID1 was subcloned into the HindIII and EcoRI sites of the pcDNA3.1Myc/His B vector using oligonucleotides 5’-CCC AAG CTT ATG TTC AGC CTG CCC-3’ and 5’-CGG GAA TTC CAG CCA TCA TCG GA-3’ to generate a plasmid expressing the PID1-6×His fusion protein. An empty expression vector (pGPU6–NC–shRNA), a PID1-silenced vector (pGPU6–PID1–shRNA), a pcDNA3.1Myc/HisB empty vector, or a PID1–pcDNA3.1Myc/His B expression vector was stably transfected into 3T3-L1 preadipocytes using Lipofectamine 2000. The stably transfected cells were grown in phenol red-free Dulbecco’s modified Eagle’s medium supplemented with 10% FBS (WISENT, CA) and 1% penicillin/streptomycin (P/S; WISENT, CA). Preadipocytes were induced to differentiate into adipocytes using a method described previously. Fully differentiated adipocytes were treated with 1 μM isoproterenol and 100 nM insulin for 24 h.

### RNAi

Differentiated adipocytes were seeded at 1.5×10^5^ cells in a 12-well plate and transfected with 75 nM AKT-siRNA or scrambled siRNA (Thermo Scientific). The targeting sequences were as follows: AKT, GAGAGGACCUUCCAUGUAG and UGCCAUUCUACAACCAGGA. After 72 h, cells were treated with 100 nM insulin and 1 μM isoproterenol for 24 h and harvested. Equal amounts of protein from different lysates were resolved by SDS-PAGE, and immunoblot analyses were performed with the indicated antibodies.

### Statistical analysis

The normality of the distribution of variables was assessed using the Kolmogorov–Smirnov test. The results are expressed as the mean±standard error of the mean (SE). Between-group differences were assessed using the *t*-test or one-way ANOVA with post hoc Bonferroni correction as appropriate. Differences were considered significant at P<0.05.

## Results

### Changes in body weight and adipose tissue weight

**Fig 1:**
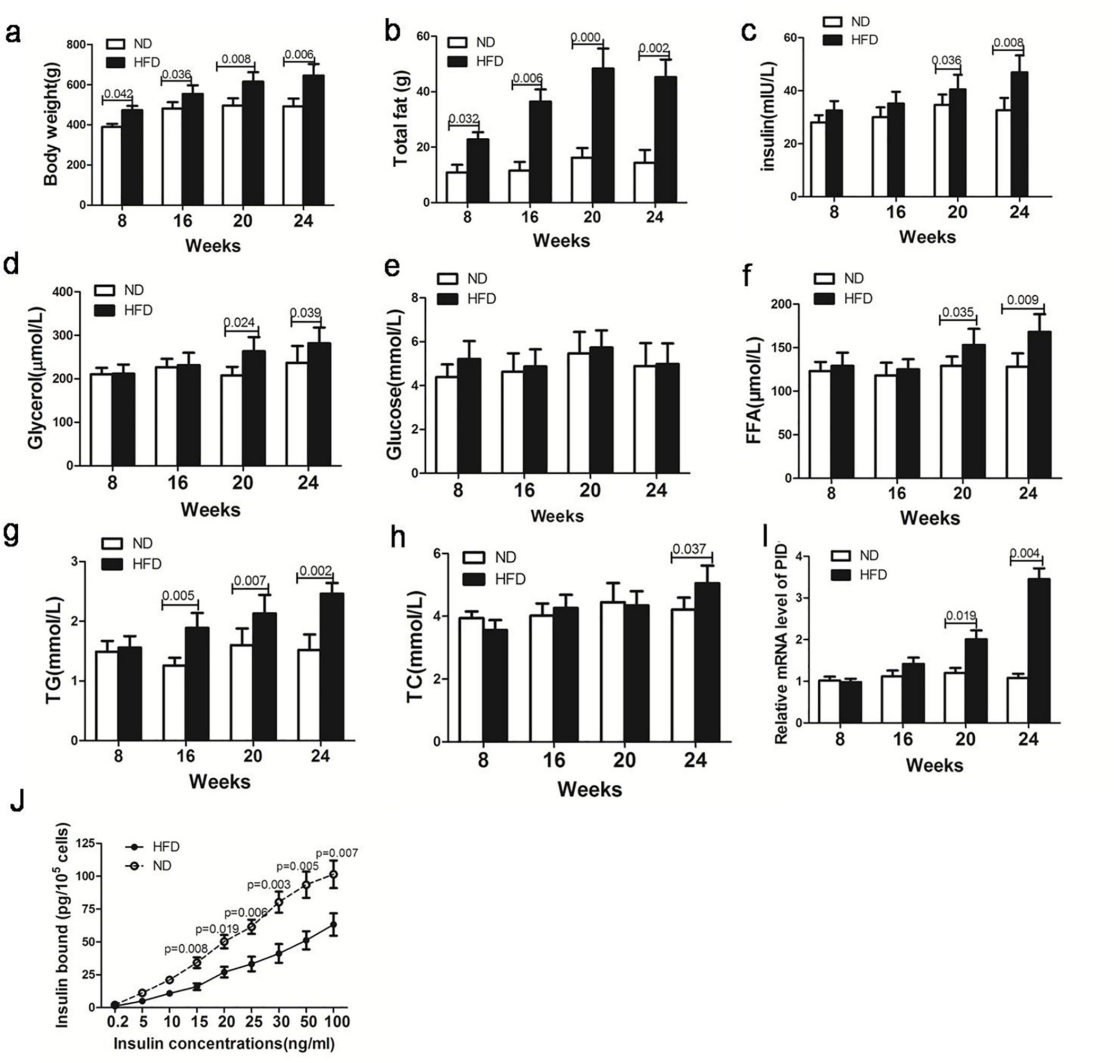
Body weight, total fat, insulin, glycerol, blood glucose, triglyceride, cholesterol and free fatty acid (FFA) levels in ninety-six Sprague-Dawley rats fed either chow (normal diet, ND) or a high-fat diet(HFD) at 8, 16, 20, and 24 weeks (eight rats from the ND group and sixteen rats from the HFD group were randomly selected at each time point). Insulin bound to the receptors of fat cells, and the expression of PID1 in white adipose tissue (WAT) was determined. (a)Body weights of rats in the two groups. (b)Total fat (perirenal and epididymal fat pads) of rats in the two groups. (c)Plasma levels of insulin in the two groups. (d) Plasma levels of glycerol in the two groups. (e) Plasma levels of glucose in the two groups. (f) Plasma levels of FFAs in the two groups. (g) Plasma levels of triglycerides in the two groups. (h) Plasma levels of cholesterol in the two groups.(I)Relative mRNA expression of PID1 in WAT of the HFD and ND groups; the control ratio was normalized to 1. (j)Insulin binding to perirenal adipocytes obtained from HFD and ND rats. Isolated fat cells were incubated with mono-^125^I-(Tyr A14) insulin with or without various concentrations of unlabeled insulin. Specific insulin binding was determined. Data are presented as the mean±SEM.

The body weight (BW) of rats in the HFD and ND groups was measured at 8, 16, 20 and 24 weeks. The initial mean BW in the 2 groups did not differ significantly. At week 8, the HFD group had a significantly higher BW than the ND group, and the BWs remained higher throughout the 24-week dietary period (Fig 1A). Similarly, after 8 weeks, the epididymal and peripheral fat depot weights in the HFD group were heavier than those in the ND group (Fig 1B), which reflected increased body fat content induced by a high-fat diet.

### Serum glucose, insulin, glycerol, triglyceride, cholesterol and FFA levels in the HFD and ND groups

After 16 weeks on the diet, the triglyceride levels for the HFD groups were significantly increased compared with the ND groups (Fig 1G). However, until 24 weeks, the cholesterol levels in the HFD group were significantly higher than those in the ND group. At 20 weeks, plasma glycerol and free fatty acid (FFA) levels in the HFD group were significantly higher than those in the ND group (Fig 1D and 1F). Similarly, at 20 weeks, the insulin levels in the HFD group were significantly higher than those in the ND group (Fig 1C). However, no significant between-group difference was observed with respect to serum glucose level throughout the 24-week dietary period (Fig 1E), suggesting a difference in the metabolic response to the diet between the 2 groups. Moreover, a good correlation between insulin and glycerol levels was observed at all time points (r = 0.57, P = 0.018). Since glycerol is an indicator of lipolysis, we hypothesized that the antilipolytic effect of insulin was impaired in HFD rats.

### Level of insulin binding to fat cells

We further measured the levels of insulin bound to receptors on fat cells in the 2 groups at 24 weeks. The results showed that adipocytes from the HFD rats bound significantly more insulin at all insulin concentrations tested. At the lowest insulin concentration used (0.2 ng/mL), insulin binding to adipocytes from HFD rats was 1.22±0.23% compared with 2.33±0.21% in adipocytes from ND rats (Fig 1J).

### mRNA expression of PID1 in white adipose tissue

We also examined the mRNA expression of PID1 in the white adipose tissue (WAT) of HFD and ND rats. At 20 weeks, the mRNA expression of PID1 in the HFD group was significantly higher than that in the ND group. At 24 weeks, PID1 mRNA expression in the HFD group was further increased compared to that at 20 weeks and was significantly higher than that in the ND group (Fig 1I). Furthermore, a positive correlation between PID1 mRNA expression and glycerol levels was observed in the 2 groups (r = 0.38, P = 0.026), suggesting that PID1 may play a role in lipolysis.

### Effect of insulin exposure on isoproterenol-stimulated lipolysis in 3T3-L1 cells

**Fig 2:**
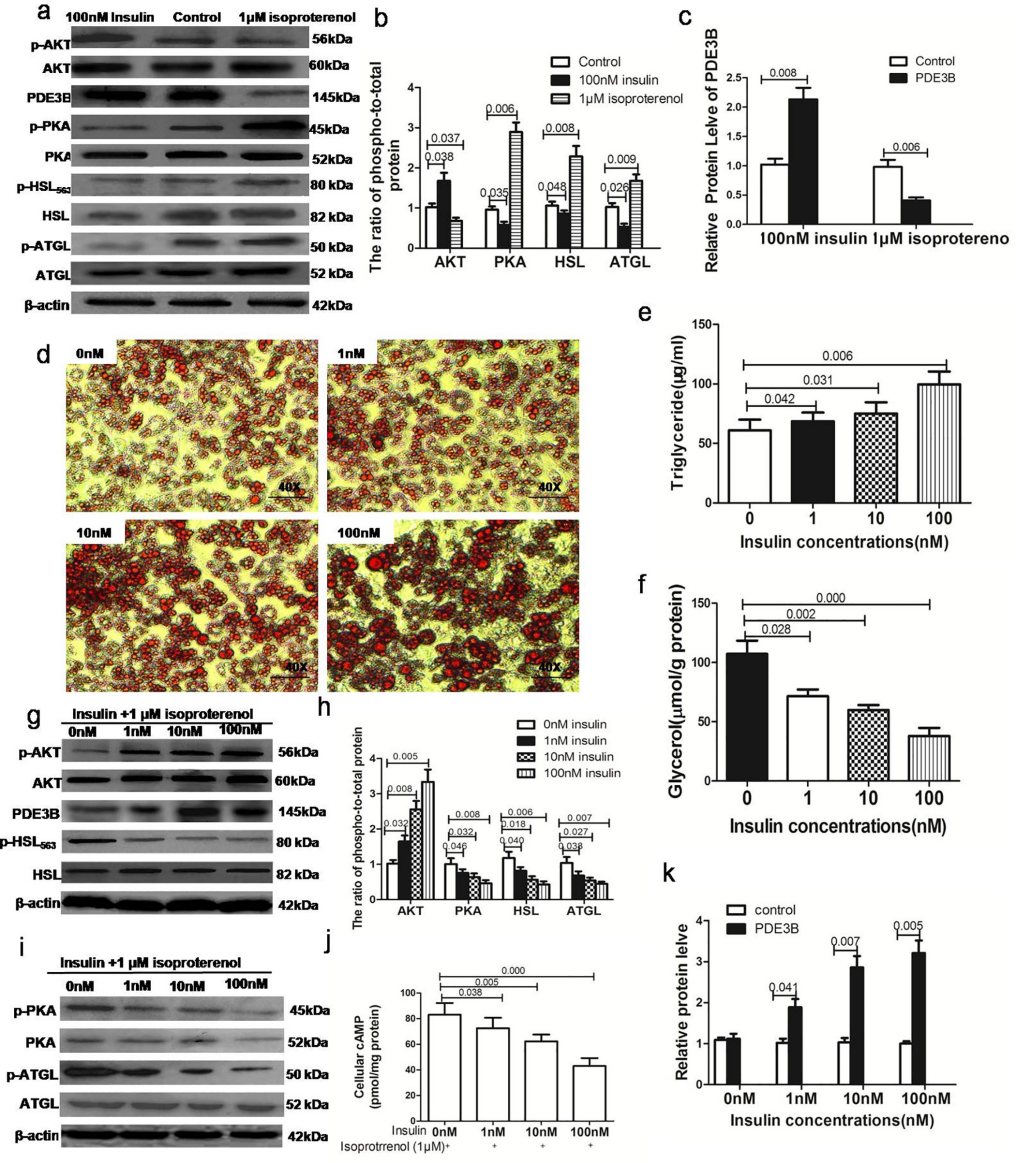
Insulin inhibits isoproterenol-stimulated lipolysis in 3T3-L1 adipocytes via the AKT/PKA/HSL pathway. We treated differentiated adipocytes with 100 nM insulin and 1 μM isoproterenoland examined the effects on lipolysis(in triplicate). (a)Western blot analyses ofAKT, PDE3B, PKA, HSL and ATGL protein levels in the insulin, isoproterenol and control groups. Phosphorylated AKT (p-AKT), phosphorylated PKA (p-PKA), phosphorylated HSL (p-HSL) and phosphorylated ATGL (p-ATGL) expression was normalized to their total protein level as a loading control. (b)The phosphorylated protein/total protein ratios of AKT, PKA and HSL were calculated, and the control ratio was normalized to 1. (c)Western blot analyses of PDE3B protein levels in the insulin, isoproterenol and control groups. Differentiated 3T3-L1 adipocytes were treated with different doses of insulin (1, 10,100 nM) and 1 μM isoproterenol for 24 h(in triplicate). (d) Cellular triglycerides were stained with Oil red O. Bar, 50 μm. (e) The amount of lipids was quantified by the Oil red O staining technique. (f)The concentration of glycerol in the medium was detected in different groups(g, i)Western blot analyses of AKT, PDE3B, PKA, HSL and ATGL protein levels in different groups. Phosphorylated AKT (p-AKT), phosphorylated PKA (p-PKA), phosphorylated HSL (p-HSL) and phosphorylated ATGL (p-ATGL) expression was normalized to their total protein level as a loading control. (h) The phosphorylated protein/total protein ratios were calculated, and the control ratio was normalized to 1.(j)cAMP levels in different groups. (k) The expression of PDE3Bwas determined in different groups by Western blot.

In contrast to the role of β-adrenergic agonists in the activation of lipolysis, insulin is a key inhibitor of lipolysis [11]. First, we separately tested the effects of isoproterenol and insulin on lipolysis in differentiated 3T3-L1 cells. As expected, insulin significantly inhibited glycerol release, while isoproterenol promoted glycerol release. In addition, PDE3B expression and AKT phosphorylation were also increased upon exposure to insulin; however, the phosphorylation of PKA and HSL was inhibited. On the other hand, the expression of the above molecules was reversed upon exposure to isoproterenol (Fig 2A–2C).

Second, we examined the effect of different doses of insulin on lipolysis induced by isoproterenol in 3T3-L1 cells. After 3T3-L1 cells were fully differentiated, the cells were treated with different doses of insulin (1, 10, 100 nM) and 1 μM isoproterenol for 24 h at 37°C; subsequently, the glycerol concentration in the cell medium was measured. Consistent with the results of previous studies [14], we found that insulin induced a concentration-dependent decrease in glycerol release, with a significant reduction observed at 100 nM insulin (Fig 2F); in addition, the intracellular lipids increased with increasing insulin dose. We used this concentration in subsequent experiments (Fig 2D and 2E).

### Insulin suppresses isoproterenol-stimulated lipolysis via phosphorylation of the AKT signaling pathway

**Fig 3:**
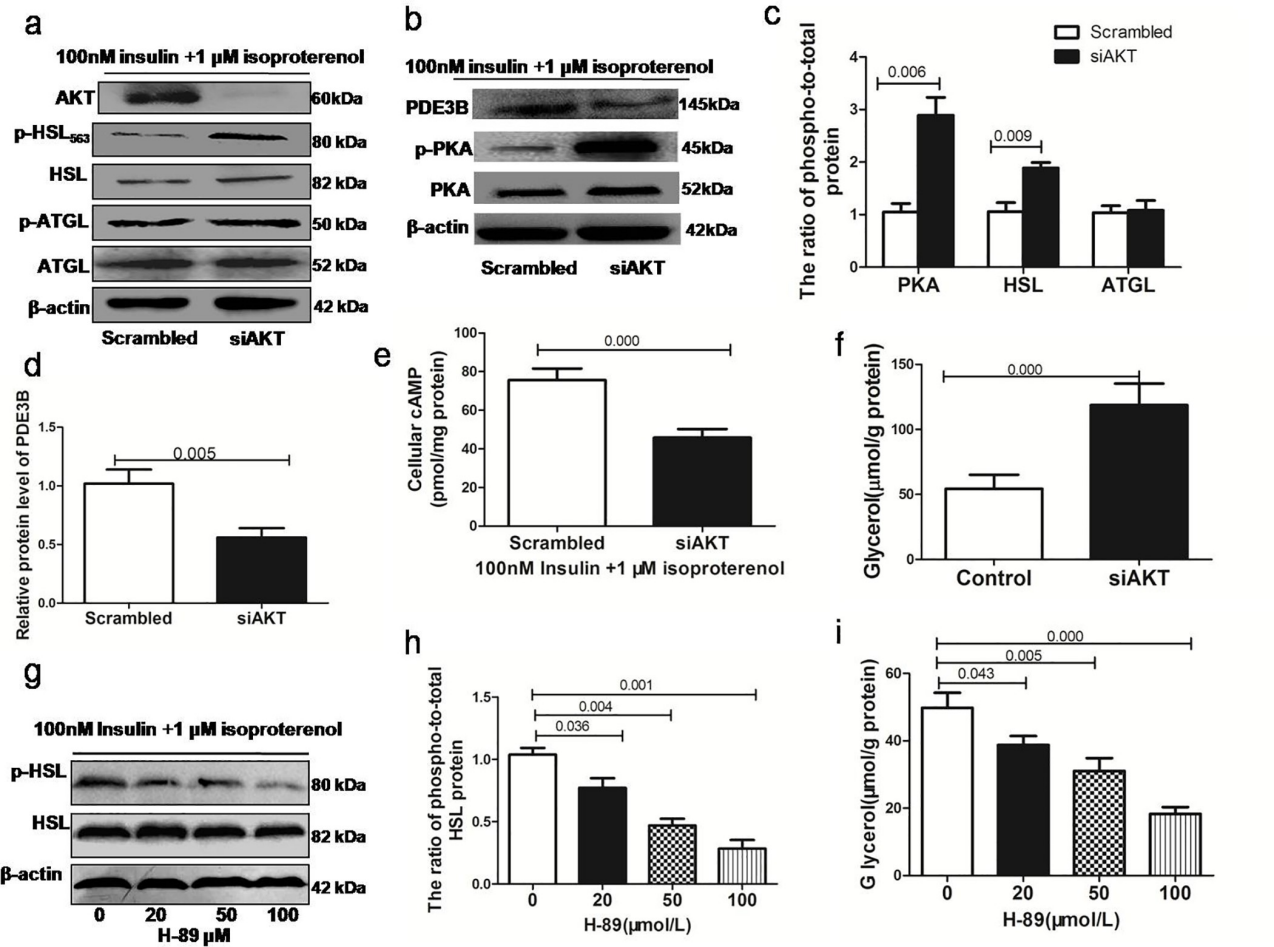
The effects of the depletion of AKT or PKA on the regulation of lipolysis by insulin. Differentiated adipocytes were transfected with AKT siRNAortreated with the PKA inhibitor and incubated in the presence of 100 nM insulin and 1 μM isoproterenol for 24 h (in triplicate). (a-b)Protein expression of PDE3B, PKA, HSL and ATGL in 3T3-L1 adipocytes transfected with AKT siRNA. Phosphorylated AKT (p-AKT), phosphorylated PKA (p-PKA), phosphorylated HSL (p-HSL) and phosphorylated ATGL (p-ATGL) expression was normalized to their total protein level as a loading control. (c-d)Relative protein expression of PDE3B and the phosphorylated protein/total protein ratios for PKA, HSL, and ATGL; the control ratio was normalized to 1. (e)Determination of cAMP levels. (f) Glycerol released into the medium after transfection with an siRNA targeting AKT. (g)Western blot analyses of HSL and phosphorylated HSL (p-HSL) protein expression in 3T3-L1 adipocytes treated with different doses of the PKA inhibitor. (h) The phosphorylated protein/total protein ratio for HSL in 3T3-L1 adipocytes treated with different doses of the PKA inhibitor. (i) Glycerol released into the medium after treatment with different doses of the PKA inhibitor. *P<0.05; **P<0.01.

We assessed whether AKT was required for the inhibitory effect of insulin on the suppression of isoproterenol-stimulated lipolysis and whether insulin inhibits isoproterenol-stimulated lipolysis by affecting the level of cAMP and PDE3B expression. We found that insulin increased AKT phosphorylation and PDE3B expression in 3T3-L1 cell lines in a dose-dependent manner. In contrast, the level of cAMP was significantly reduced with increasing insulin concentrations (Fig 2G–2K). Furthermore, differentiated adipocytes were transfected with AKT siRNA and incubated in the presence of 100 nM insulin and 1 μM isoproterenol for 24 h, and we examined the effects of AKT knockdown on PDE3B expression, cAMP levels and glycerol release in 3T3-L1 cells. AKT siRNA transfection led to >80% knockdown of the target genes, decreased PDE3B expression and increased cAMP levels and glycerol release (Fig 3A–3F). These results suggest that the antilipolytic effect of insulin on 3T3-L1 adipocytes is mediated by AKT.

### Insulin suppresses isoproterenol-stimulated lipolysis via the decreased phosphorylation of PKA and HSL

The suppression of lipolysis by insulin involves the activation of PDE3B by AKT, which leads to a decrease in PKA and the subsequent inactivation of HSL. Our results confirmed that the antilipolytic effect of insulin is mediated by AKT; therefore, we further assessed whether insulin inhibits isoproterenol-stimulated lipolysis by affecting the phosphorylation of PKA and HSL. After the addition of different doses of insulin and 1 μM isoproterenol for 24 h, we analyzed the phosphorylation of HSL at its major PKA site and the phosphorylation of PKA. We observed a significant decrease in the phosphorylation levels of PKA and HSL upon exposure to insulin (Fig 2G, 2I and 2H). On the other hand, AKT siRNA promoted the phosphorylation of HSL and PKA after treatment with insulin and isoproterenol (Fig 3A–3C). Western blot analysis showed that pretreatment with PKA inhibitors also caused a dose-dependent decrease in HSL phosphorylation and the release of glycerol by 3T3-L1 cells (Fig 3G–3I). These data confirm that insulin-induced AKT phosphorylation results in the dephosphorylation of PKA and HSL and suppresses isoproterenol-stimulated glycerol release by 3T3-L1 cells.

The recently discovered adipose triglyceride lipase (ATGL) does not seem to be involved in the catecholamine resistance of lipolysis observed in abdominal subcutaneous adipose tissue of obese subjects [15]. Interestingly, we showed that ATGL phosphorylation (similar to HSL phosphorylation) is suppressed in 3T3-L1 adipocytes by different doses of insulin (1, 10, 100 nM) and 1 μM isoproterenol. At a dose of 100 nM, insulin significantly reduced ATGL phosphorylation to 45% at 24 h. However, AKT siRNA transfection did not affect ATGL expression or phosphorylation. These findings indicate that insulin-mediated ATGL downregulation is independent of the AKT signaling pathway (Fig 3A and 3C).

### Effects of PID1 on lipolysis and phosphorylation of the AKT/PKA/HSL signaling pathway and ATGL molecules

**Fig 4:**
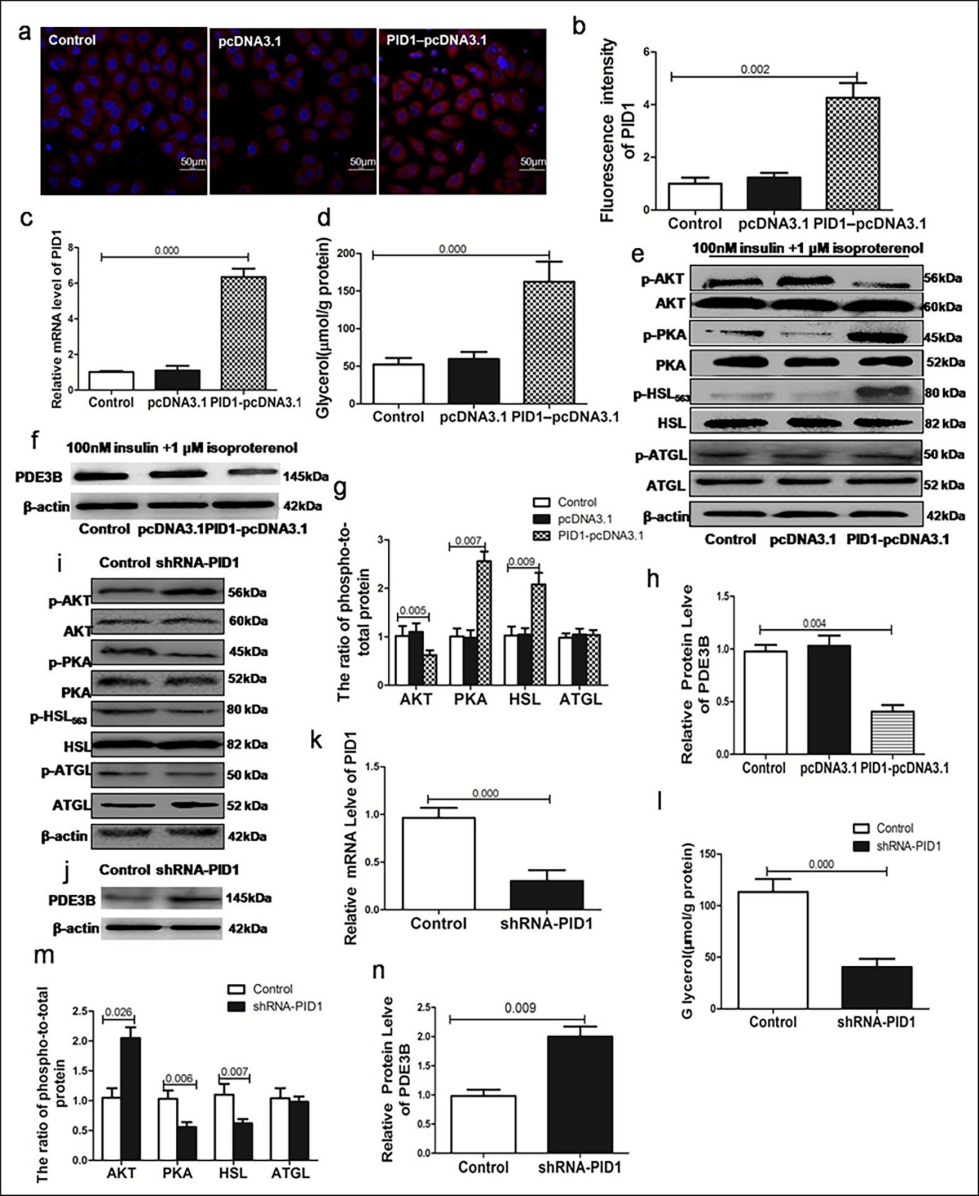
Effects of PID1 expression on lipolysis and the phosphorylation of AKT/PDE3B/PKA/HSL signaling molecules and ATGL. Preadipocytes were subjected to PID1knockout or upregulation and allowed to differentiate into 3T3-L1 adipocytes; these cells were treated with 1 μM isoproterenol and 100 nM insulin for 24 h(in triplicate). (a-b)Immunofluorescence analysis was performed to assess the expression of the PID1 gene in empty vector cells, PID1-overexpressing cells, and control cells.(c)RT-PCR analyses of the mRNA expression of PID1 in empty vector cells, PID1-overexpressing cells, and control cells. (d)Glycerol released into the medium after the upregulation of PID1.(e-f)Protein expression of AKT, PDE3B, PKA, HSL and ATGL in empty vector cells, PID1-overexpressing cells, and control cells. Phosphorylated AKT (p-AKT), phosphorylated PKA (p-PKA), phosphorylated HSL (p-HSL) and phosphorylated ATGL (p-ATGL) expression was normalized to their total protein level as a loading control.(g)The phosphorylated protein/total protein ratios for AKT, PKA, HSL, and ATGL in 3T3-L1 adipocytes after transfection with the PID1 overexpression plasmid. (h)The expression of PDE3B was determined by Western blot after the PID1 overexpression plasmid. (i-j)Protein expression of AKT, PDE3B, PKA, HSL and ATGL after transfection with PID1 shRNA. Phosphorylated AKT (p-AKT), phosphorylated PKA (p-PKA), phosphorylated HSL (p-HSL) and phosphorylated ATGL (p-ATGL) expression was normalized to their total protein level as a loading control. (k)RT-PCR analyses of mRNA after transfection with PID1 shRNA. (l) Glycerol was released into the medium after knockdown of PID1. (m)Phosphorylated protein/total proteinratios for AKT, PKA, HSL, and ATGL after PID1 knockdown. (n)The expression of PDE3B was determined by Western blot after transfection with PID1 shRNA.

We sought to determine the underlying mechanism by which PID1 affected lipolysis and whether this mechanism was involved in the AKT/PKA/HSL signaling pathway. Preadipocytes were transfected with PID1 plasmids and allowed to differentiate; the differentiated 3T3-L1 adipocytes were treated with 1 μM isoproterenol and 100 nM insulin for 24 h. The expression of PID1 was verified by immunofluorescence and RT-PCR (Fig 4A–4C). We found that glycerol release in cells overexpressing PID1 was approximately 2 fold higher than that in control cells (Fig 4D). Additionally, PID1 overexpression resulted in a noticeable inhibition of AKT phosphorylation and PDE3B expression in the presence of insulin (Fig 4E–4H). We also evaluated the phosphorylation of PKA and HSL, downstream signaling molecules of AKT in the insulin antilipolytic signaling pathway. We found that PKA and HSL phosphorylation levels were significantly increased in cells overexpressing PID1 (Fig 4E–4H). We also evaluated the phosphorylation of ATGL and found no significant change in the phosphorylation of ATGL (Fig 4E–4H). We further explored whether PID1 knockdown could reverse the antilipolytic effect of insulin. As shown in Fig 4I–4N, PID1 knockdown increased AKT phosphorylation and PDE3B expression and resulted in a noticeable inhibition of cAMP levels and the phosphorylation of PKA and HSL, which in turn led to the inhibition of isoproterenol-stimulated glycerol release in 3T3-L1 cells. However, PID1 knockout did not affect the phosphorylation of ATGL. This result indicates that PID1 promotes lipolysis in the presence of insulin, which involves the inhibition of AKT phosphorylation and the phosphorylation of PKA and HSL.

## Discussion

Our animal experiments showed that the mRNA levels of PID1 in adipose tissue from HFD rats were higher than those in adipose tissue from ND rats at 20 weeks and showed a positive correlation with insulin levels and lipolysis in blood. Mechanistically, in 3T3-L1 adipocytes, we found that the antilipolytic effect of insulin is mediated by AKT and that AKT phosphorylation by insulin can result in the inhibition of PDE3B expression and an elevation of cAMP levels, which in turn leads to the dephosphorylation of PKA and HSL and the suppression of glycerol release. In addition, it is possible that, as has been demonstrated for HSL, some degree of ATGL phosphorylation occurs to coordinately regulate triglyceride hydrolysis. Isoproterenol stimulation results in ATGL phosphorylation, but in contrast to HSL, this modification are apparently not mediated by AKT. However, the overexpression of PID1 and treatment with 1 μM isoproterenol and 100 nM insulin for 24h resulted in an increased release of glycerol and a noticeable inhibition of AKT phosphorylation, PDE3B expression and the phosphorylation of PKA/HSL in 3T3-L1 cells but did not affect the phosphorylation of ATGL.

High-fat diets have long been used to study fat storage, insulin sensitivity, and glucose tolerance and metabolism in experimental animals. In general, diets containing 60% fat and 20% carbohydrate for 4–8 weeks resulted in an approximately 40–50% increase in body weight, fat stores and insulin level compared to a normal diet. However, there was no significant difference with respect to the plasma levels of glucose or FFAs [16–17]. In these models, we believe that FFA levels were not elevated because the duration of high-fat feeding was not long enough. We extended the duration of the high-fat diet and found that a long-term high-fat diet led to the accumulation of visceral fat and an increase in plasma insulin and FFA levels.

The levels of circulating FFAs depend primarily on the rates of lipolysis in adipose tissue. One of the key physiological functions of insulin as the major anabolic hormone in the body is to restrain lipolysis and promote fat storage in adipose tissue in the postprandial state [18, 19]. Insulin, the most important physiological inhibitor of catecholamine-induced lipolysis, was shown to induce the phosphorylation and activation of phosphodiesterase type 3B (PDE3B), which led to a decrease in PKA and HSL activity [20]. In our in vitro experiments, we found that insulin inhibited isoproterenol-induced lipolysis in a dose-dependent manner, in parallel with the decreased release of glycerol, promotion of AKT phosphorylation and PDE3B expression, and dephosphorylation of PKA, HSL and ATGL. Pretreatment with PKA inhibitors also caused a dose-dependent decrease in HSL expression and glycerol release in 3T3-L1 cells. These results indicate that insulin inhibits lipolysis not only by dephosphorylating HSL but also by reducing ATGL phosphorylation. Our findings are consistent with those of previous studies. However, whether the insulin-induced inhibition of lipolysis depends on AKT is not clear. Findings from experimental studies suggest that the insulin-induced inhibition of lipolysis involves the phosphorylation of AKT, which in turn leads to the phosphorylation of PDE3B, thus stimulating lipolysis [21, 22]. Other studies suggest that insulin antagonizes triglyceride hydrolysis via a mechanism that is independent of AKT [23]. We showed that AKT depletion inhibited PDE3B expression, activated PKA and HSL phosphorylation, and ameliorated the inhibitory effect of insulin on lipolysis, but AKT depletion had no effect on ATGL phosphorylation. These data confirm that AKT is essential for the antilipolytic effect of insulin and that insulin modulates AKT activity via phosphorylation, but these results also show that the phosphorylation of ATGL is not required for AKT.

A wide body of evidence has implicated a defect in antilipolysis as the critical etiological abnormality that initiates the positive amplifying circuit that characterizes insulin resistance [24–26]. However, the molecular mechanism of the impaired control of lipolysis in obesity has yet to be elucidated.

Many studies have indicated that PID1 may play an important role in the development of obesity-related IR [27, 28]. Zhao et al found that PID1 can impair insulin signal transduction [29]. In adipocytes and muscle cells, PID1 also inhibits the insulin-mediated phosphorylation of insulin receptor substrate-1(IRS-1) and the insulin-mediated translocation of the GLUT-4 glucose transporter, which results in decreased glucose uptake [30, 9]. PID1−/− mice exhibited improved glucose tolerance and insulin sensitivity under a chow diet, with increased AKT phosphorylation in WAT [31].Therefore, we hypothesized that PID1 may impair the phosphorylation of insulin signaling molecules that inhibit lipolysis. Many studies have demonstrated increased expression of PID1 in adipose tissues in obesity [32]. In the present study, we also found increased mRNA expression of PID1 in adipose tissue from HFD rats. Moreover, our results show that PID1 promoted lipolysis and induced a noticeable inhibition of AKT phosphorylation and PDE3B expression and the phosphorylation of PKA/HSL in 3T3-L1 cells but did not affect the phosphorylation of ATGL.

Since this study merely tested lipolysis in the presence of insulin, further studies on the expression of PID1 and the phosphorylation of AKT in 3T3-L1 cells without insulin treatment are needed to determine whether PID1 expression and AKT phosphorylation are dependent on insulin. In addition, this study did not conclusively show that the inhibition of AKT and induction of the phosphorylation of PKA and HSL are the mechanisms by which PID1 promotes lipolysis. Proof of this mechanism awaits the generation and analysis of the appropriate loss- and gain-of-function mediators downstream of PID1using in vivo models.

## Conclusion

**Fig 5:**
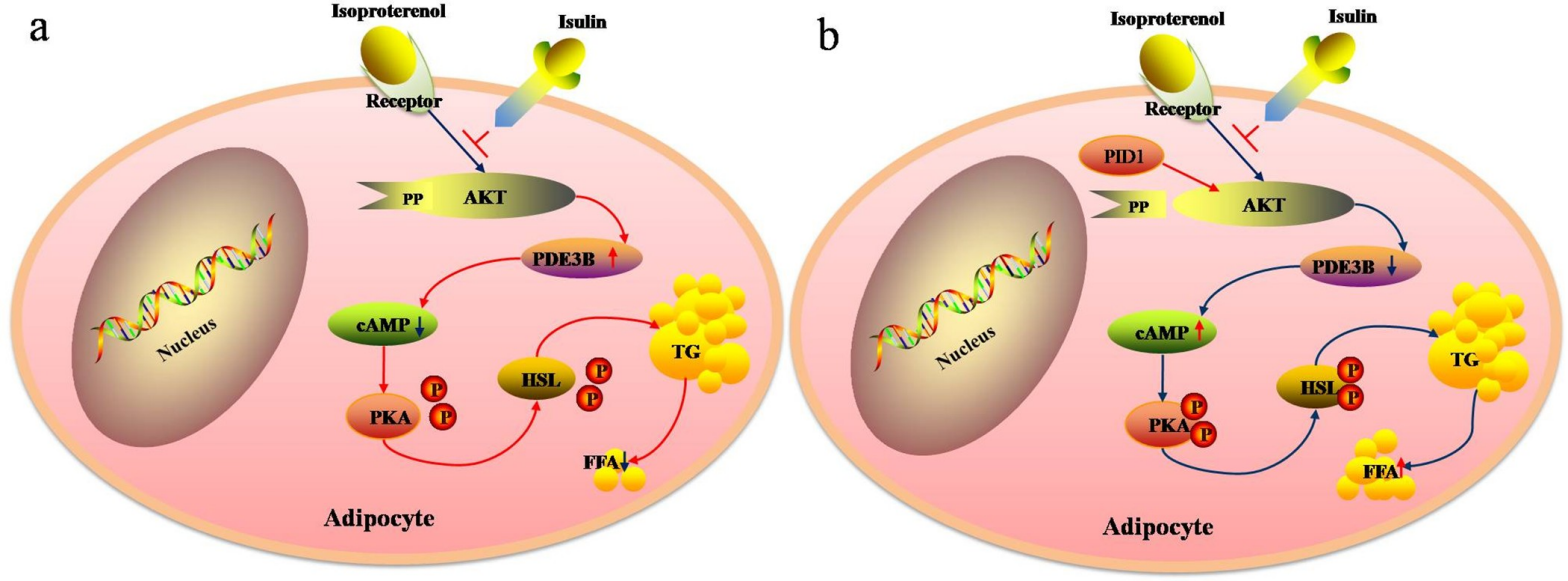
A hypothetical model showing how PID1 promotes lipolysis by regulating the AKT/PDE3B/PKA/HSL signaling pathway, which is supported by the results of this study. (a)Insulin inhibits isoproterenol-induced lipolysis by increasing PDE3B expression via AKT. The elevation of PDE3B catalyzes the hydrolysis of cAMP, which reduces the cellular level of cAMP. The lowering of cAMP further dephosphorylates PKA and thereby results in a decrease in hormone-sensitive lipase (HSL) and lipolysis. (b)PID1 promotes lipolysis and induces a noticeable inhibition of the phosphorylationof AKT and PDE3B expression, which further increases cAMP levels and the phosphorylation of PKA and HSL, leading to increased lipolysis.

In conclusion, our results demonstrate that PID1 promotes lipolysis in the presence of insulin, which is mediated via the inhibition of AKT phosphorylation and PDE3B expression and the phosphorylation of PKA and HSL (Fig 5). These findings provide new insights into the mechanisms of lipolysis in obesity. These findings may lead to new therapeutic avenues to ameliorate adipocyte lipolysis and improve insulin sensitivity.

## Supporting information

S1 Data (RAR)
