# Correction: PID1 alters the antilipolytic action of insulin and increases lipolysis via inhibition of AKT/PKA pathway activation

**DOI:** 10.1371/journal.pone.0218721

**Published:** 2019-06-17

**Authors:** Chunyan Yin, Wei hua Liu, Yuesheng Liu, Li Wang, Yanfeng Xiao

Fig 4 is incorrect. Please see the correct Fig 4 here.

**Fig 4:**
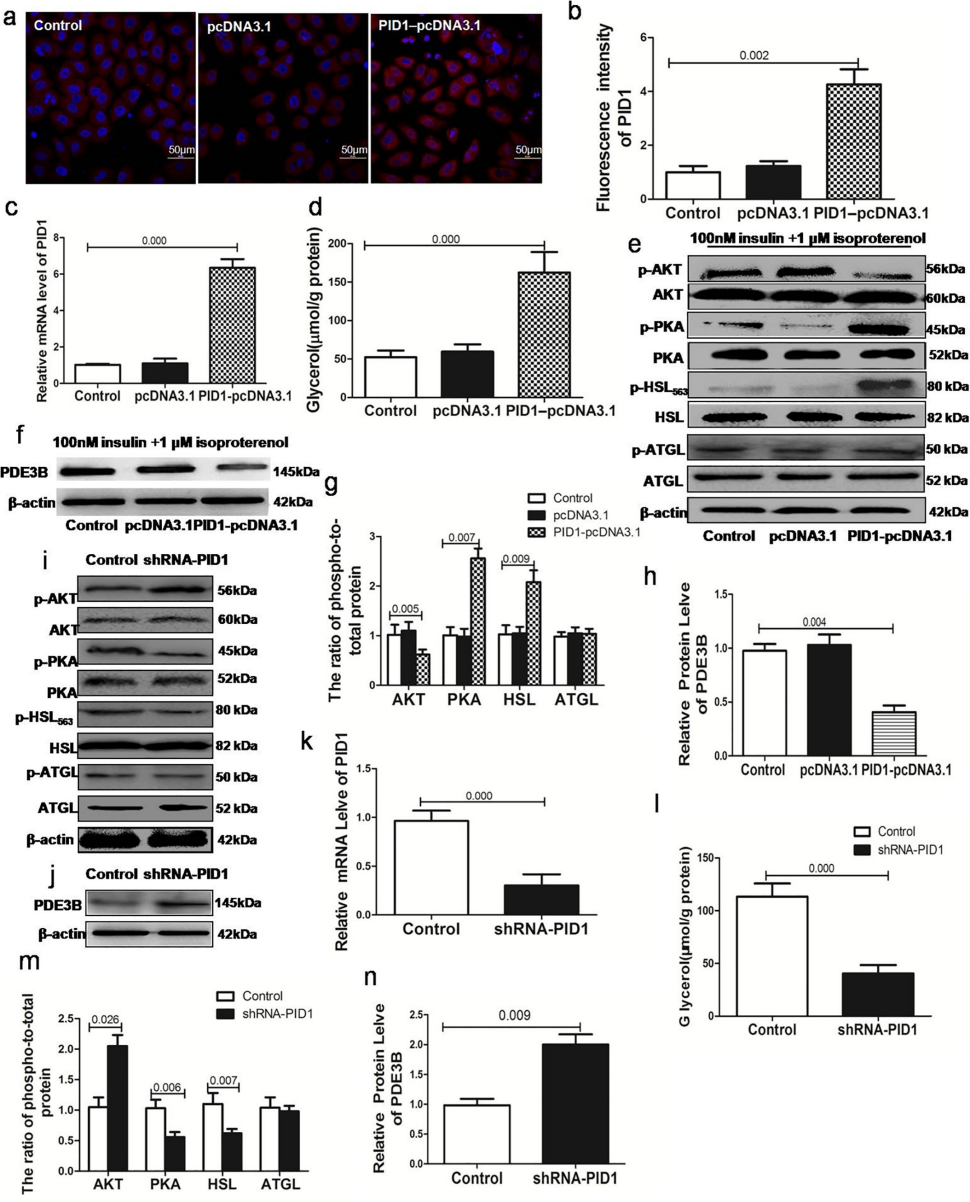
Effects of PID1 expression on lipolysis and the phosphorylation of AKT/PDE3B/PKA/HSL signaling molecules and ATGL. Preadipocytes were subjected to PID1knockout or upregulation and allowed to differentiate into 3T3-L1 adipocytes; these cells were treated with 1 μM isoproterenol and 100 nM insulin for 24 h(in triplicate). (a-b)Immunofluorescence analysis was performed to assess the expression of the PID1 gene in empty vector cells, PID1-overexpressing cells, and control cells.(c)RT-PCR analyses of the mRNA expression of PID1 in empty vector cells, PID1-overexpressing cells, and control cells. (d)Glycerol released into the medium after the upregulation of PID1.(e-f)Protein expression of AKT, PDE3B, PKA, HSL and ATGL in empty vector cells, PID1-overexpressing cells, and control cells. Phosphorylated AKT (p-AKT), phosphorylated PKA (p-PKA), phosphorylated HSL (p-HSL) and phosphorylated ATGL (p-ATGL) expression was normalized to their total protein level as a loading control.(g)The phosphorylated protein/total protein ratios for AKT, PKA, HSL, and ATGL in 3T3-L1 adipocytes after transfection with the PID1 overexpression plasmid. (h)The expression of PDE3B was determined by Western blot after the PID1 overexpression plasmid. (i-j)Protein expression of AKT, PDE3B, PKA, HSL and ATGL after transfection with PID1 shRNA. Phosphorylated AKT (p-AKT), phosphorylated PKA (p-PKA), phosphorylated HSL (p-HSL) and phosphorylated ATGL (p-ATGL) expression was normalized to their total protein level as a loading control. (k)RT-PCR analyses of mRNA after transfection with PID1 shRNA. (l) Glycerol was released into the medium after knockdown of PID1. (m)Phosphorylated protein/total proteinratios for AKT, PKA, HSL, and ATGL after PID1 knockdown. (n)The expression of PDE3B was determined by Western blot after transfection with PID1 shRNA.
